# A trend of Korean older adults’ awareness as a threat to society during the COVID-19 pandemic

**DOI:** 10.1093/geroni/igab046.3651

**Published:** 2021-12-17

**Authors:** Soonhyung Kwon, Jaesung An, Oejin Shin

**Affiliations:** 1 University of Illinois at Urbana-Champaign, Savoy, Illinois, United States; 2 California State University East Bay, Hayward, California, United States; 3 University of Illinois at Urbana Champaign, Savoy, Illinois, United States

## Abstract

**Background:**

Unlike ageism that consists of one’s prejudice, stereotype, and discrimination toward older adults, age-based threats are one’s negative cognition (Levy, 2001). Previous studies indicated that the younger generation stigmatizes the older generation as unworthy during the COVID-19 pandemic (Meisner, 2021). However, there is no study looking at how older adults perceive themselves as threats to society during this time of the pandemic. Thus, our study aimed to understand the varying trend of older adults’ awareness as a threat to society in association with socio-economic profiles before and during the pandemic. Method: This study included 637 Korean older adults who answered the older generation’s threats to society from 2018 to 2020. We used Latent Class Analysis (LCA) to categorize participants into different subgroups that shared distinct patterns of threats to society. Multinomial logistic regression examined how the subgroups in threats to society were associated with socio-demographic characteristics in each year.

**Results:**

For three waves, three clusters of threats to society (low, mid, and high) were identified. Although the mid-level of threat remained the same (60% of the sample for three years), the high level of threats has been doubled in 2019 (25%) compared to 2018 (11%) and 2020 (13%). Regarding the associated socio-demographic characteristics with threats to society, those who being female in 2018 and younger age in 2020 were more likely to be associated with mid-level of threats to society.

**Discussion:**

Further study needs to identify the relationship between awareness as a threat to society and health outcomes.

